# Evaluation of Welded Lap Joints Using Ultrasonic Guided Waves

**DOI:** 10.3390/s24051384

**Published:** 2024-02-21

**Authors:** Hussain Altammar, Mohammad Faseeulla Khan

**Affiliations:** Department of Mechanical Engineering, College of Engineering, King Faisal University, Al Ahsa 31982, Saudi Arabia; fmohammad@kfu.edu.sa

**Keywords:** ultrasonic waves, welded joints, damage identification, steel plates, disbond, corrosion

## Abstract

Welded lap joints play a vital role in a wide range of engineering structures such as pipelines, storage tanks, pressure vessels, and ship hulls. This study aims to investigate the propagation of ultrasonic guided waves in steel welded lap joints for the baseline-free inspection of joint defects using the mode conversion of Lamb waves. The finite element method was used to simulate a single lap joint with common defects such as corrosion and disbonding. To identify the propagating wave modes, a wavenumber–frequency analysis was conducted using the 2D fast Fourier transform. The power loss of the transmitted modes was also determined to identify damage in the lap joints. The results indicate that the A0 incident in pristine conditions experienced significant transmission losses of about 9.5 dB compared to an attenuation of 2.8 dB for the S0 incident. The presence of corrosion was found to reduce these transmission losses by more than 28%. In contrast, introducing disbonding in the lap joint increased the transmission loss of the S0 incident, while a negligible loss was observed for the A0 incident. The mode-converted S0 (MC-S) and mode-converted A0 (MC-A0) incidents were found to exhibit a unique sensitivity to the presence of corrosion and disbonding. The results indicate that MC-S0 and MC-A0 as well as Lamb mode incidents interact differently in terms of corrosion and disbonding, providing a means to identify damage without relying on baseline signals.

## 1. Introduction

Lap joints are widely used across various industries, such as the aerospace, automotive, and oil and gas industries, as a means of joining structural components. These joints play a crucial role in ensuring the optimal performance of the assembled parts. However, the presence of defects within lap joints can significantly compromise their strength and integrity [1,2,3,4,5]. In recent years, ultrasonic guided waves (UGWs) have emerged as a powerful technique for the non-destructive testing and evaluation of lap joints [6,7,8]. This technique can assess large areas to identify hidden defects while providing valuable information about the size, location, and type of these defects. By analyzing the characteristics of guided waves, including their velocity, amplitude, and dispersion behavior, it is possible to obtain detailed information about the characteristics of existing defects in lap joints.

Disbonding and corrosion are commonly observed defects in welded lap joints. The formation of such defects involves different mechanisms and processes. Disbonding typically occurs due to welding issues, resulting in insufficient bonding between the base metal and the weld metal. The presence of disbonding greatly compromises the structural integrity of the joint, leading to a decreased load-carrying capacity and increased susceptibility to fatigue failure [9,10]. On the other hand, corrosion can occur due to environmental factors, such as exposure to moisture or corrosive substances, and can lead to the weakening and thinning of the joint material [11]. Ongoing research still focuses on the identification of these defects using ultrasonic guided waves [12,13,14].

The presence of defects, such as corrosion and disbonding, can cause mode conversion and generate additional wave modes [15]. These modes can significantly affect the signals received during inspection and complicate defect characterization. On the other hand, they can also be used to correlate scattered modes with certain defects which could aid in baseline-free inspections. Ong et al. [16] investigated the scattered modes of fundamental guided waves in a pitch/catch arrangement to develop a baseline-free inspection technique for the detection of disbonding in adhesive lap joints. Their findings revealed that the presence of the A0 mode in the received signals strongly indicated the presence of disbonding in lap joints when using excitation frequencies ranging from 300 kHz to 400 kHz. Salmone et al. [17] presented a structural health monitoring approach for lap joints using autoregressive models to analyze the received signals and estimate the severity of disbonding. In another study conducted by Fasel and Todd [18], the focus was on investigating disbonding in composite bonded joints using transducers mounted on the surface in a pitch-catch scheme. It was found that an excitation signal at a center frequency of 300 kHz provided the best assessment of the bonded joints’ integrity.

The study of Lamb wave propagation through lap joints is a topic of significant research interest [19,20,21,22]. Understanding how Lamb waves interact with lap joints and defects within these joints is crucial to ensuring the integrity and reliability of joined structures. Jankauskas et al. [23] investigated the propagation of the symmetric Lamb mode (S0) through welded lap joints in stainless-steel plates of floor tanks. Their results demonstrate that the transmission losses of the S0 mode were influenced by the actuation frequency and the overlap width. The presence of corrosion in the overlap region was found to increase the amplitude of the transmitted S0 mode.

Chaboty et al. [24] focused on the propagation of the fundamental symmetric mode (S0) and shear horizontal mode (SH0) through welded lap joints. The objective was to identify the most promising mode and actuation frequency for a corrosion inspection of multiple lap joints in steel plates welded together in a single lap configuration. The findings revealed that the SH0 mode exhibited lower transmission losses compared to the S0 mode, enabling longer-distance travel. Fromme et al. [25] designed an array consisting of 32 piezoelectric transducers that were permanently affixed to a steel plate for structural health monitoring. They used the first antisymmetric mode A0 to detect simulated corrosion. These studies provide insights into defect characterization, emphasizing the efficacy of ultrasonic guided waves in nondestructive testing and structural health monitoring.

Additionally, the A0 Lamb mode has been frequently excluded from welded lap joint studies due to its dispersive nature and high sensitivity to the surrounding medium, limiting its long-distance travel capability [23,24]. However, several studies concerned with adhesive joints have presented promising results for the antisymmetric mode for the evaluation of adhesive joints. Nagy [26] evaluated the integrity of the adhesive bond line through the excitation of symmetric and antisymmetric waves. The sensitivity of the antisymmetric mode to joint defects was found to be higher compared to the sensitivity of the symmetric wave mode. Another study carried out by Kundu [27] examined the first seven Lamb modes. It was found that the first antisymmetric mode (A0) is the most suited to assess the integrity of the bond line. This suggests that the A0 Lamb mode could be a reliable indicator of bond line integrity in adhesive joints.

Based on a review of the literature, it is evident that despite a relatively large body of work investigating the propagation of guided waves in lap joints, most research has been concerned with adhesively bonded lap joints with little attention given to welded lap joints. Additionally, the A0 Lamb mode has been frequently excluded from welded lap joint studies due to its dispersive nature and high sensitivity to the surrounding medium, although it has been proven to be effective for identifying flaws in adhesive joints. The baseline-free inspection approach of welded lap joints is of great interest in the field of nondestructive evaluations. However, most of these research attempts considered simple structures or adhesive single lap joints [28,29].

Therefore, the objective of this study is to investigate the characteristics of fundamental Lamb modes in steel welded lap joints for a baseline-free evaluation of common joint defects, including corrosion and disbonding. The scattering and mode conversion of guided waves resulting from welded lap joints were analyzed in both pitch-catch and pulse-echo configurations using a validated finite element model. A set of waveform signals received upstream and downstream of the lap joints were processed using a 2D Fourier transform to identify the propagating wave modes in the structure. The power loss of the transmitted modes was determined to detect joint defects and identify their type without relying on baseline signals.

## 2. Materials and Methods

### 2.1. Dispersion Curves

When Lamb waves propagate through plates over a wide range of frequencies, multiple symmetric and antisymmetric wave modes can coexist and interact constructively or destructively. This behavior depends on the frequency–thickness product and material properties. In this study, the phase velocity and group velocity dispersion curves for a 1 mm thick steel plate were calculated using a software product developed by [30]. The accuracy of the software code was validated for computing dispersion curves and mode shapes of ultrasonic guided waves in isotropic plates and multilayered anisotropic laminates.

**Figure 1:**
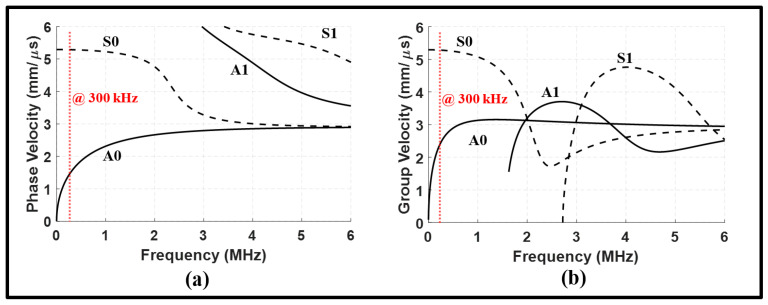
Guided wave dispersion curves for a 1 mm thick steel plate: (**a**) phase velocity dispersion curves; (**b**) group velocity dispersion curves. The velocities at 300 kHz are marked.

**Figure 2:**
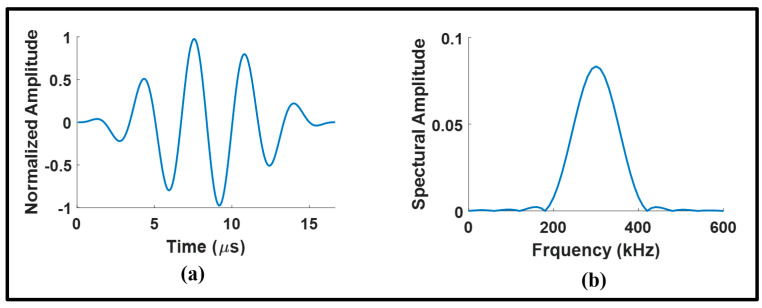
Amplitude of Hann windowed 5-cycle excitation signal at 300 kHz in (**a**) time domain and (**b**) frequency domain.

The dispersion curves of ultrasonic guided waves are presented in Figure 1. In this figure, a vertical dotted line indicates the velocity of fundamental guided waves at the operating frequency. The operating frequency corresponds to the center frequency of the excitation signals, which consists of a five-cycle Hann windowed tone burst ranging from 200 kHz to 400 kHz as shown in Figure 2. This center frequency was specifically chosen for the comparison of our results with those of existing works that have identified this frequency as an optimal center frequency for detecting joint defects [16,31,32].

**Figure 3:**
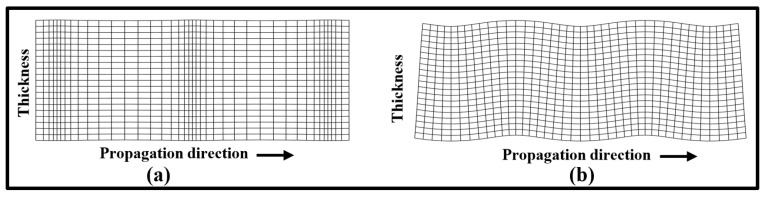
Fundamental mode shapes across 1 mm thick steel plate at 300 kHz: (**a**) symmetric mode; (**b**) antisymmetric mode.

The mode shapes of fundamental guided waves at the operating frequency are plotted in Figure 3. The symmetric mode (S0) demonstrates the particle motion aligned with the propagation direction in the *x*-axis. Conversely, the antisymmetric mode (A0) exhibits flexural behavior with lateral motion perpendicular to the wave propagation direction within the thickness of the plate.

### 2.2. Numerical Model

**Figure 4:**
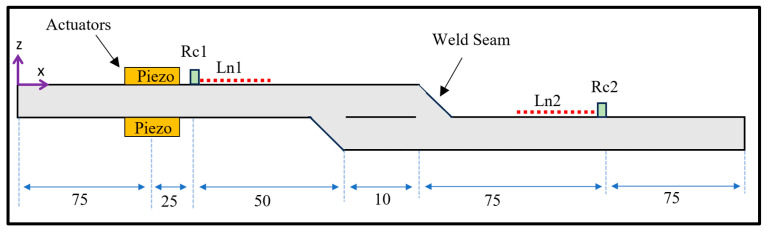
Schematic diagram for two 1 mm thick steel plates welded together by a lap joint at 45° with two actuators attached on both sides of the upper steel plate. Dimensions in mm.

Figure 4 illustrates two steel plates, measuring 160 mm × 1 mm, with an overlapping zone of 10 mm. The steel plates are joined by fillet welds at a 45° angle creating a single lap joint. The wave propagation of guided waves in the lap joint was simulated in a finite element software product, ANSYS 22.0, using a transient dynamic analysis. The model was governed by the general equations of motion in a matrix form as follows [33]:

(1) $$\left[M\right]\left\{\ddot{u}\right\}+\left[C\right]\left\{\dot{u}\right\}+\left[K\right]\left\{u\right\}=\left\{F\left(t\right)\right\}$$

where [*M*] is the structural mass matrix; [*C*] is the structural damping matrix; [*K*] is the structural stiffness matrix; {*F*(*t*)} is the vector of the applied nodal loads; and {*u*}, {$\dot{u}$}, and {*ü*} are the nodal displacement and its time derivatives, respectively. For the sake of simplicity and without loss of generality, the structural damping was set to zero. The equations of motion were solved by the Newmark time-integration method. The model was meshed by a higher-order two-dimensional PLANE183 element with eight nodes, two degrees of freedom at each node, and translations in the nodal x and y directions. To ensure accurate modeling, the model was meshed with a minimum of 40 elements per the smallest wavelength [34]. A fine mesh was also implemented at the weld regions. The numerical simulation has a total of 25,954 elements, including 20,558 solid elements and 5396 contact elements. The material properties of steel plates defined in the model were as follows: modulus of elasticity E = 200 GPa, modulus of rigidity G = 80 GPa, Poisson’s ratio ν = 0.3, and density ρ = 7850 kg/m^3^. The interface region between the plates was kept free to allow frictional interaction between the two plates. The time step was set to 0.075 μs to provide adequate temporal and spatial resolution of the fastest propagating mode in the medium.

Piezoelectric actuators with a length of 10 mm were incorporated into the upper substrate using the pin-force model. A time-varying longitudinal displacement was applied to the nodes whose the actuators were defined. Symmetric and antisymmetric modes were excited through the expansion and contraction of the predefined region. Specifically, the symmetric mode was actuated by applying identical signals to the predefined nodes at the top and bottom surface of the upper substrate. On the other hand, excitation signals of the same amplitude but opposite in value were applied to the predefined nodes to actuate antisymmetric mode. This approach is widely adopted and experimentally validated in numerous studies [35,36,37]. More comprehensive information about this approach is available in [38].

Waveform signals were obtained from two sensing points as depicted in Figure 4. The first sensing point, Rc1, was positioned before the weld lap joint. The waveform signals were acquired by selecting a set of nodes at a distance of 100 mm from the origin. The second sensing point, Rc2, was situated after the lap joint at a distance of 235 mm. This arrangement was chosen to investigate the transmission of guided wave modes through the joint while minimizing signal overlap between mode incidents and converting modes. Sufficient time was allowed for the guided wave modes to propagate separately.

To identify guided wave modes, the two-dimensional Fast Fourier Transform (2D FFT) was employed. Since the mode shapes of S0 and A0 waves reside in the same plane, the 2D FFT is particularly beneficial. By performing a 2D Fourier transform on the received signals, the wave amplitude H(k,f) can be obtained as a function of wavenumber and frequency [39]:

(2) $$H\left(k,f\right)=\int_{-\infty }^{+\infty }\int_{-\infty }^{+\infty }\varepsilon \left(x,t\right)e^{-i\left(kx+\omega t\right)}dxdt$$

where ε(x,t) is the normal strain signal on the surface of the plate in the x direction at time t, ω=2πf is the angular frequency, k=ω/c is the wavenumber, and c denotes the phase velocity. In this study, the method involves monitoring the received signals from two lines on the surface of plate over a specified period. In Figure 4, the upstream and downstream monitoring lines were defined by acquiring the average elemental strain over the lines denoted as Ln1 and Ln2. These measurements were captured over the predefined regions with equidistant increments to ensure an adequate amount of spatial data for dispersive analysis. The power loss was determined through the lap joint as follows:

(3) $$\alpha =-20\log\left(\frac{P_{down}}{P_{up}}\right)$$

where $P_{up}$ is the amplitude of the monitored mode measured upstream of the lap joint using 2D FFT, and $P_{down}$ is the amplitude of the monitored mode measured downstream of the lap joint using 2D FFT.

## 3. Results and Discussion

### 3.1. Validation of Numerical Mode

**Figure 5:**
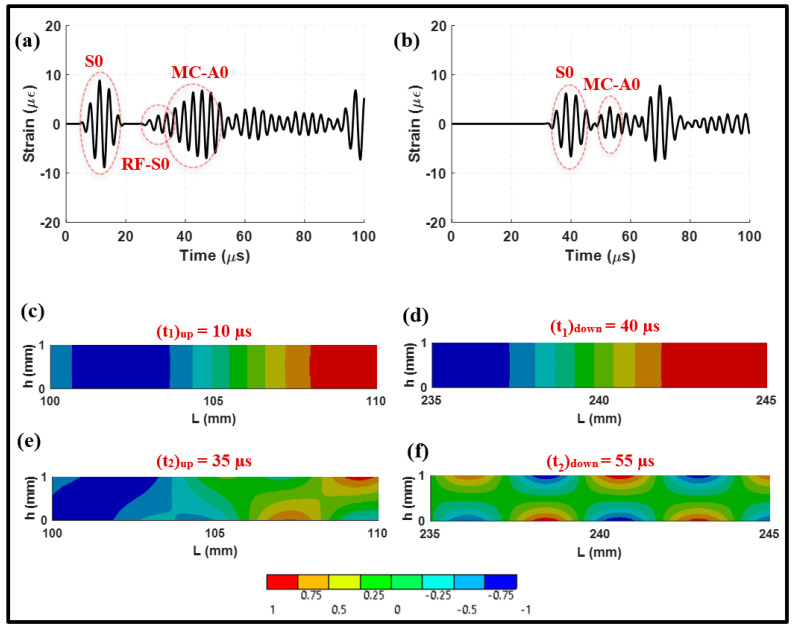
Received waveform signals from symmetric actuation at (**a**) 100 mm, upstream; and (**b**) 235 mm, downstream. Two-dimensional full-field distribution of elastic waves for normal strain in the *x*-direction at (**c**) 10 μs, upstream; (**d**) 40 μs, downstream; (**e**) 35 μs, upstream; and (**f**) 55 μs, downstream.

This section presents a validation of the finite element model for wave modes propagating in steel plates in pristine conditions. The symmetric mode S0 was excited by applying a five-cycle Hann windowed tone burst with a center frequency of 300 kHz to the top and bottom piezoelectric transducers. To examine the propagation of wave modes, the signals captured before and after the lap joint are displayed in Figure 5a,b. The mode shapes of normal elastic waves corresponding to the main wave packets are presented Figure 5c–f. In Figure 5a, the initial arrival in the upstream signal represents the mode incident wave which maintains its shape with some amount of attenuation due to transmission through the lap joint. Table 1 includes the group velocities and power losses of the S0 incident and MC-A0 in pristine conditions. The group velocity of the S0 incident was determined to be 5.3 mm/μs, which is in agreement with the predicted group velocity using the dispersion curves in Figure 1. There is less than a 3% difference between the numerical and analytical group velocities.

The power loss of the S0 incident is estimated at 2.89 dB. In a previous study [23], the power loss of the S0 mode transmitted through a weld lap joint was numerically predicted and experimentally validated to be about 4.3 dB. The lower power loss predicted by the numerical model herein can be attributed to the presence of a weld seam at each edge of the lap joint, which allows for the smoother transmission of the propagating waves.

Furthermore, Figure 5c,d provides the full-field distributions of the elastic waves in the *x*-direction. These distributions reveal the mode shapes of the propagating waves before and after the lap joint. They exhibit a uniform normal strain across the width which closely resembles that determined for the S0 mode in Figure 3a. Upon closer examination, the downstream signal at 55 μs exhibits maximal strain at the top and bottom surfaces of the plate, but zero strain at the neutral axis. This observation supports the presence of the fundamental A0 mode in the medium as depicted in Figure 3b. It is worth noting that previous studies, including numerical and experimental investigations [16,23,24], have consistently demonstrated this behavior. These results validate numerical models employed in simulating guided waves in steel plates.

### 3.2. Guided Waves in Pristine Conditions

#### 3.2.1. Actuation of Symmetric Mode

In this section, a more comprehensive analysis of Figure 5 is provided. The signals collected from the upstream and downstream regions represent the pulse-echo and pitch-catch arrangements, respectively. Figure 5a,b demonstrate a mode conversion of the S0 incident into the A0 mode. The converted A0 mode which originated from the S0 incident is referred to as MC-A0. It is observed that the S0 incident propagating in the *x*-direction interacts with the 45° weld, resulting in the A0 mode. MC-A0 can reflect off and transmit through the lap joint. Thus, it can be identified in the upstream and downstream signals. Figure 5a shows the S0 incident followed by its reflection from the joint. This mode is referred to as RF-S0. The multimode wave packet at 35 μs consists of the RF-S0 and MC-A0 modes. On the other hand, the mode incident in Figure 5b is directly followed by MC-A0 as can be seen from the mode shape at 55 μs (see Figure 5f). It can be concluded that the lap joint serves as the source of the MC-A0 mode, which is emitted inwards and outwards from the joint. The group velocity was determined at 2.51 mm/μs using the received signals from the sensing points. It should be noted that the remaining wave packets in Figure 5a,b correspond to reflections from the boundaries.

**Figure 6:**
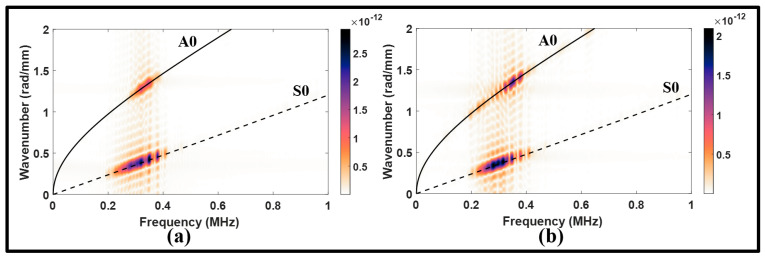
Wavenumber–frequency distributions from symmetric actuation for: (**a**) upstream of the lap joint, pristine; (**b**) downstream of the lap joint, pristine.

The 2D FFT was employed to 80 normal strain signals captured at equidistant points of 0.775 mm on the surface of the plates. The upstream and downstream monitoring lines were initiated at 80 mm and 170 mm. By superimposing the analytical dispersion curves of the S0 and A0 modes onto the wavenumber–frequency distributions, the scattered modes and their frequency ranges can be identified. Figure 6 illustrates the wavenumber–frequency distributions of the S0 incident before and after the lap joint. The results indicate the presence of the S0 and A0 modes on both sides of the lap joint. While the mode incident experienced a slight attenuation of 2.89 dB, this loss transformed into a gain for the MC-A0 in the downstream signal as can be seen in Figure 6. Furthermore, the interaction of guided waves with the lap joint introduced sporadic peaks that span over a wide range of wavenumbers. These peaks are scattered and the superposed modes are attributed the presence of the welded lap joint.

#### 3.2.2. Actuation of Antisymmetric Mode

**Figure 7:**
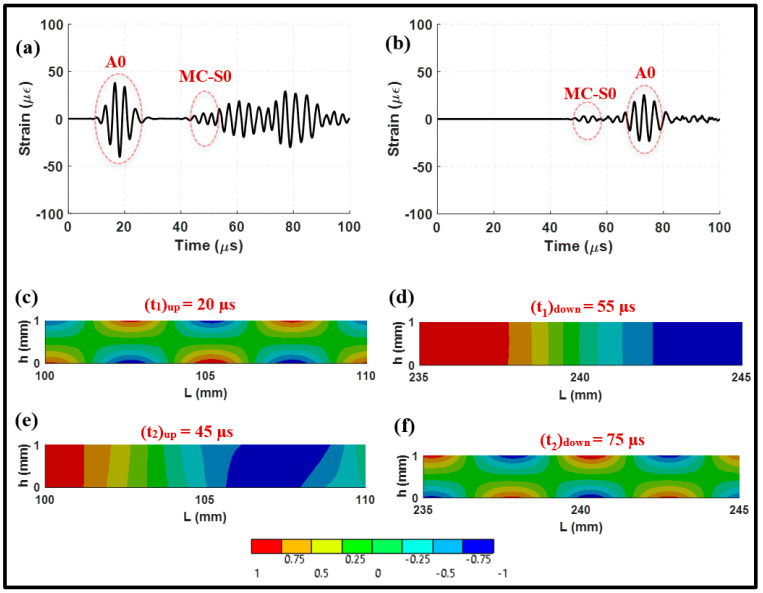
Received waveform signals from antisymmetric actuation at (**a**) 100 mm, upstream; and (**b**) 235 mm, downstream. Two-dimensional full-field distribution of elastic waves for normal strain in the *x*-direction at (**c**) 20 μs, upstream; (**d**) 55 μs, downstream; (**e**) 45 μs, upstream; and (**f**) 75 μs, downstream.

Figure 7 shows the waveform signals and mode shapes of the A0 incident and converted modes. The received signals upstream and downstream of the lap joint are presented in Figure 7a,b. In the upstream signal, it can be observed that the A0 incident arrives first, followed by the mode-converted S0 initiated at the lap joint. The mode-converted S0 which originated from the A0 incident is referred to as MC-S0. The presence of MC-S0 in the upstream signal makes it challenging to track and identify this mode accurately in practical scenarios. However, the downstream signal provides a higher resolution, with wave modes existing separately. The separation of modes makes the pitch-catch configuration more attractive for weld joint inspections. It is important to note that the A0 incident suffers noticeable transmission losses. However, these losses can be mitigated by increasing the amplitude of actuation. The A0 mode is practically easier to actuate, offering a potential solution to counteract the transmission losses encountered.

The mode shape captured at 45 μs of the upstream signal is shown in Figure 7e. This time instant marks the arrival of the reflected A0 mode, which overlaps with the MC-S0 mode. This time frame allows us to observe the complex interaction between these modes. To further validate the presence and characteristics of fundamental wave modes within the medium, the group velocities of these modes were calculated. The values were found to be comparable to the analytical solutions, as summarized in Table 2.

**Figure 8:**
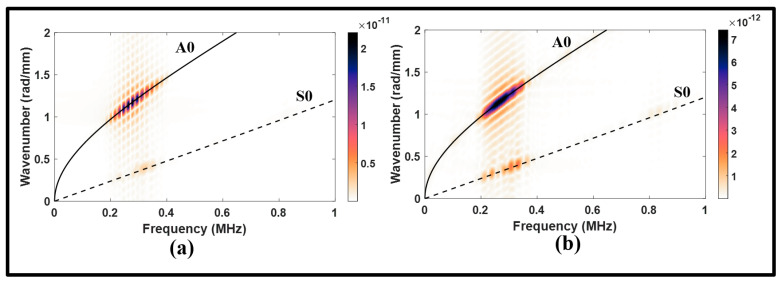
Wavenumber–frequency distributions from antisymmetric actuation for: (**a**) upstream of the lap joint, pristine; and (**b**) downstream of the lap joint, pristine.

A wavenumber–frequency analysis was conducted to assess the amplitudes of propagating modes over the monitoring lines using a 2D FFT technique. Figure 8 clearly illustrates that the A0 incident dominates in both the upstream and downstream distributions, although it undergoes a significant attenuation upon transmission through the joint with a power loss of about 9.46 dB. It can be noted that the presence of the S0 mode is negligible in the upstream and downstream signals. Consequently, it can be inferred that a pitch–catch arrangement can yield improved resolution signals when employing an antisymmetric actuation. It is worth noting that the A0 incident experiences notable transmission losses in contrast to the S0 incident, which is consistent with previous studies highlighting the behavior of the A0 incident to exhibit higher transmission losses than those of the S0 incident [40].

### 3.3. Effects of Corrosion on Guided Waves

#### 3.3.1. Actuation of Symmetric Mode

The formation of an oxide layer resulting from corrosion can lead to a weak bonding of lap joints at the interface. Therefore, a bonded contact approach was employed to simulate this corrosion in a welded lap joint and prevent any separation or sliding at the plate interface. This simple simulation technique proved to be effective in representing the corrosion within joints [23].

**Figure 9:**
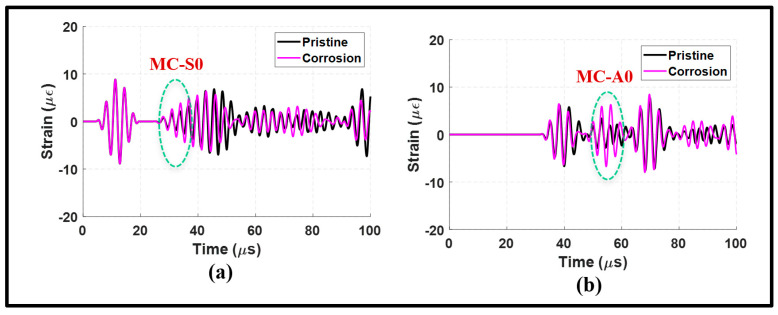
Symmetric actuation with corrosion at the lap joint: (**a**) upstream signals at 100 mm; and (**b**) downstream signals captured at 235 mm.

**Figure 10:**
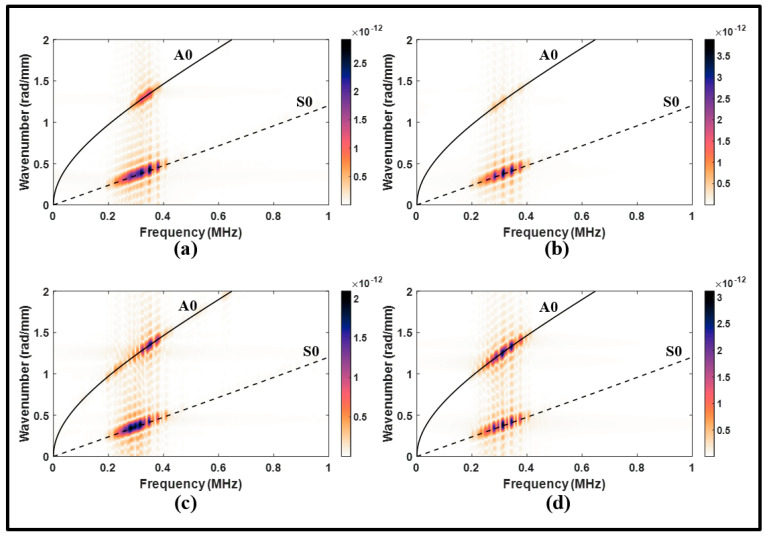
Wavenumber–frequency distributions from symmetric actuation for (**a**) upstream of the lap joint, pristine; (**b**) upstream of the lap joint, corrosion; (**c**) downstream of the lap joint, pristine; and (**d**) downstream of the lap joint, corrosion.

To analyze the waveform signals captured in both pristine and corrosion conditions, sensing points were placed upstream and downstream of the lap joint. The results, as depicted in Figure 9, revealed multiple reflections and mode conversions, indicating that relying on the baseline signal to inspect corrosion in a pulse-echo configuration might lead to false-negative results. On the other hand, corrosion noticeably reduced the transmission losses of the MC-A0 mode. This suggests that employing a pitch-catch arrangement could offer higher resolution signals. This effect is demonstrated in the downstream signals shown in Figure 9b. In Figure 10a,b, the frequency analysis is conducted along the upstream and downstream lines. It can be observed that the presence of corrosion resulted in a reduction in the transmission losses of the MC-A0 mode in comparison to those in the pristine conditions. These findings were further supported by the wavenumber–frequency analysis in Figure 10c,d. This is also consistent with previous studies that demonstrated positive gain on the transmitted modes through the lap joints [23].

#### 3.3.2. Actuation of Antisymmetric Mode

**Figure 11:**
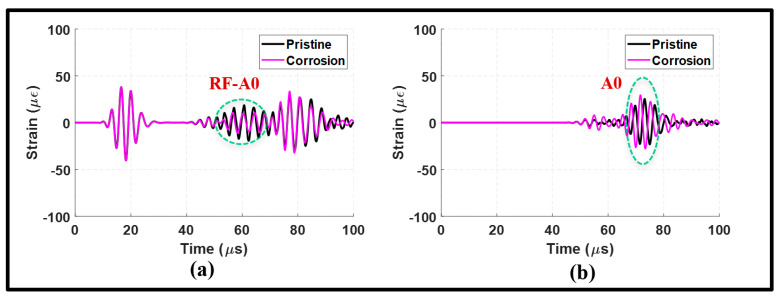
Antisymmetric actuation with corrosion at the lap joint: (**a**) upstream signals at 100 mm; and (**b**) downstream signals at 235 mm.

This analysis focuses on an antisymmetric actuation with corrosion introduced at the welded lap joint. The results in Figure 11 reveal that the A0 mode is the dominant mode in the upstream and downstream signals. The signals obtained from the antisymmetric actuation exhibit significantly enhanced the resolution in comparison to the symmetric actuation. This suggests that using the A0 incident to determine corrosion has the potential to reduce the complexity of signal processing and minimize false alarms.

**Figure 12:**
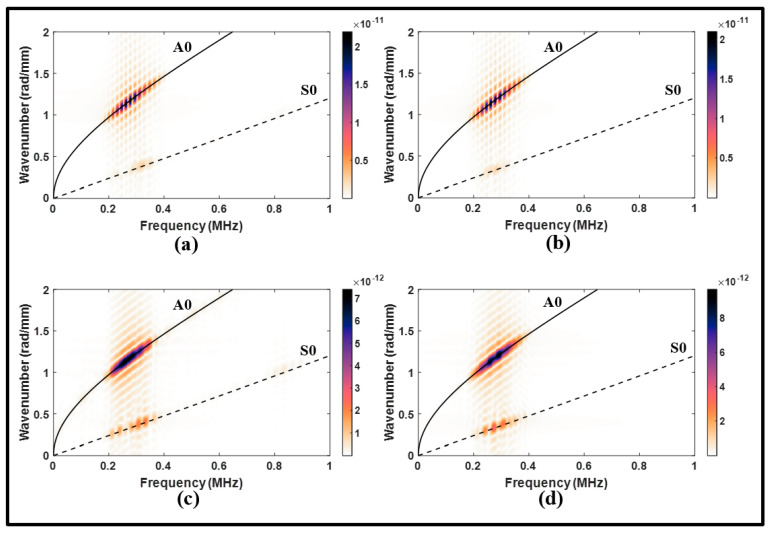
Wavenumber–frequency distributions from antisymmetric actuation for (**a**) upstream of the lap joint, pristine; (**b**) upstream of the lap joint, corrosion; (**c**) downstream of the lap joint, pristine; and (**d**) downstream of the lap joint, corrosion.

Figure 11a illustrates some distortion in the propagating modes observed from the upstream signals. It remains, however, relatively minor when compared to the pristine signal. Conversely, corrosion has led to an increase in the amplitude of the transmitted A0 incident as shown in Figure 11b. The presence of corrosion has also caused a phase shift in the A0 incident. The wavenumber–frequency analysis presented in Figure 12 further supports the dominance of the A0 mode in the presence of corrosion.

To summarize, the results depicted in Figure 11 and Figure 12 underscore the benefits of employing an antisymmetric actuation. Using a pitch-catch configuration with the A0 incident can enhance the resolution and improve signal interpretation.

### 3.4. Effects of Disbonding on Guided Waves

#### 3.4.1. Actuation of Symmetric Mode

**Figure 13:**
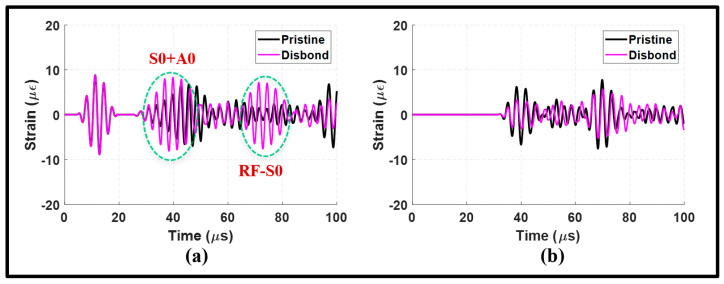
Symmetric actuation with disbonding at the lap joint: (**a**) upstream signals at 100 mm; and (**b**) downstream signals at 235 mm.

**Figure 14:**
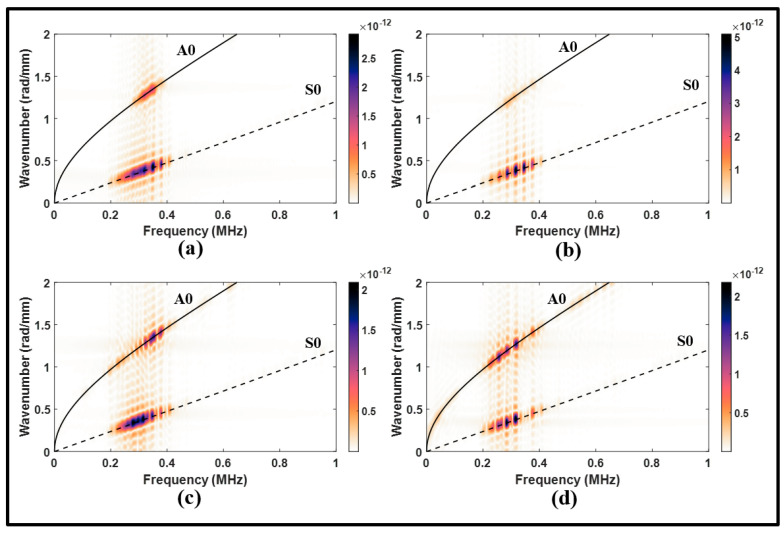
Wavenumber–frequency distributions from symmetric actuation for (**a**) upstream of the lap joint, pristine; (**b**) upstream of the lap joint, disbonding; (**c**) downstream of the lap joint, pristine; and (**d**) downstream of the lap joint, disbonding.

Simulated disbonding was introduced at the right shoulder of the lap joint by separating the two steel plates while ensuring that the other weld seam remained intact. The waveforms and wavenumber–frequency spectra obtained from the symmetric actuation are presented in Figure 13 and Figure 14.

Figure 13a clearly illustrates the significant impact of disbonding on the upstream signal. The presence of disbonding led to a multimode wave packet of RF-S0 and MC-A0, which exhibit an increased strength compared to that of the pristine state signal. Additionally, a distinct wave packet that is absent in the pristine state signal emerged. This wave packet is believed to be the reflection of the S0 incident from the right edge of the upper plate, given the absence of a transmission path due to disbonding.

Examining the wavenumber–frequency spectrums of the upstream signals in Figure 14a,b reveals that the S0 mode experienced a considerable amplification, approximately twice the amplitude of the S0 mode observed in the pristine state spectrums. Although the A0 mode in Figure 14b appears to be relatively weaker in comparison to the S0 mode, it still exhibits some strength.

Conversely, the downstream signals in Figure 13b demonstrate an attenuation in the transmitted modes of the S0 incident and MC-A0. The disbonding significantly affects the S0 incident, leading to a notable transmission loss, as can be observed from frequency spectrums of the downstream signals in Figure 14c,d. These results indicate that the disbonding effect can be observed in the upstream and downstream signals. However, unique converted modes are observed in the upstream signal, making the pulse-echo arrangement potentially more attractive to determine disbonding using the S0 incident.

#### 3.4.2. Actuation of Antisymmetric Mode

**Figure 15:**
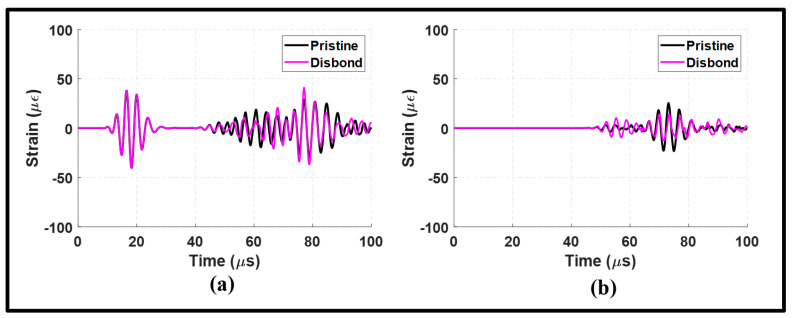
Antisymmetric actuation with disbonding at the lap joint: (**a**) upstream signals at 100 mm; and (**b**) downstream signals at 235 mm.

**Figure 16:**
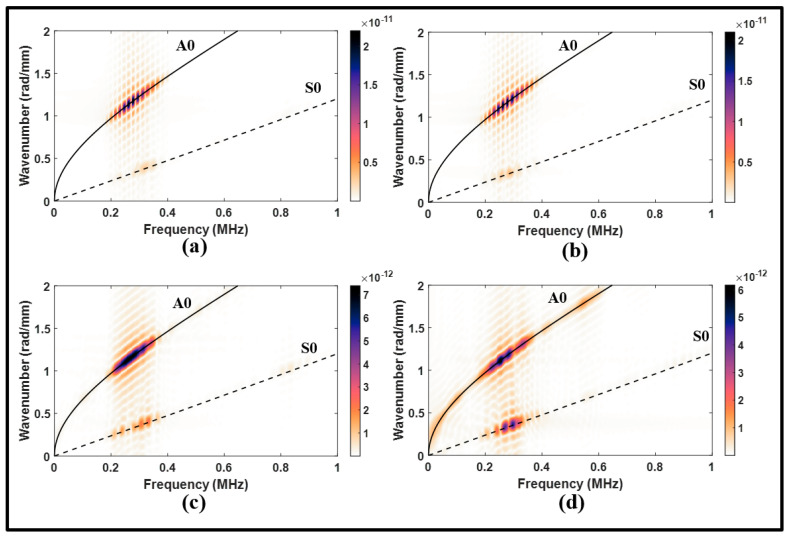
Wavenumber–frequency distributions from antisymmetric actuation for (**a**) upstream of the lap joint, pristine; (**b**) upstream of the lap joint, disbonding; (**c**) downstream of the lap joint, pristine; and (**d**) downstream of the lap joint, disbonding.

The lap joint with disbonding was also subjected to an A0 incident to examine its effects on Lamb waves. In Figure 15a, the waveform signal received from the upstream of the lap joint is similar to that observed in the pristine state. However, the reflected waves exhibited an increase in amplitude due to the presence of disbonding. The disbonding had a relatively small impact on the upstream signal, and this can be confirmed by the frequency spectrums in Figure 16a,b.

On the other hand, the received downstream signal revealed a significant amount of distortion in the MC-S0 and the transmitted A0 modes, as shown in Figure 15b. This distortion was characterized by a noticeable attenuation and phase shift. It can be noted from the wavenumber–frequency spectrum in Figure 16c,d that disbonding resulted in a wide range of frequencies along the A0 dispersion curve. These observations indicate the pitch-catch configuration is suitable to determine disbonding using the A0 incident. However, the S0 incident is found to be better suited to determine disbonding in a pulse-echo configuration.

### 3.5. Baseline-Free Inspection

**Figure 17:**
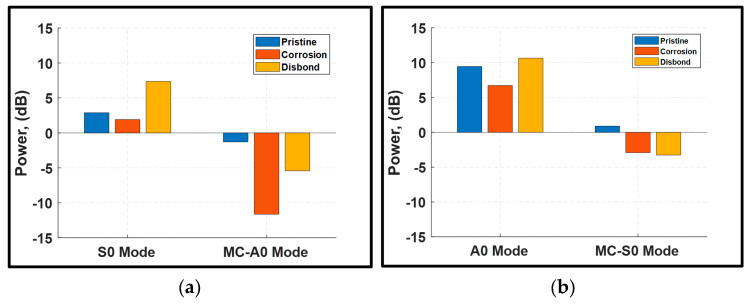
Power losses of Lamb mode incidents and converted modes using a five-cycle Hann windowed excitation signal at 300 kHz center frequency: (**a**) S0 incident; and (**b**) A0 incident.

The power losses of the transmitted and converted modes at different actuation frequencies were calculated using the 2D FFT of the upstream and downstream signals. Figure 17 presents the power losses obtained from the pristine, corrosion, and disbonding conditions by exciting fundamental Lamb modes with a five-peak Hann windowed tone burst signal at a center frequency of 300 kHz. It is important to note that pristine conditions were not involved in the calculations of power losses. However, they were included in the analysis to explore distinct trends associated with the presence of damage. Figure 17a demonstrates that the existence of corrosion or disbonding can be identified by monitoring MC-A0 when the S0 incident occurs. A noticeable gain in the range of 5 dB to 11 dB was recorded for MC-A0 in corrosion and disbonding conditions, respectively. These gains of MC-A0 represent more than four times the gain obtained from the pristine condition.

**Figure 18:**
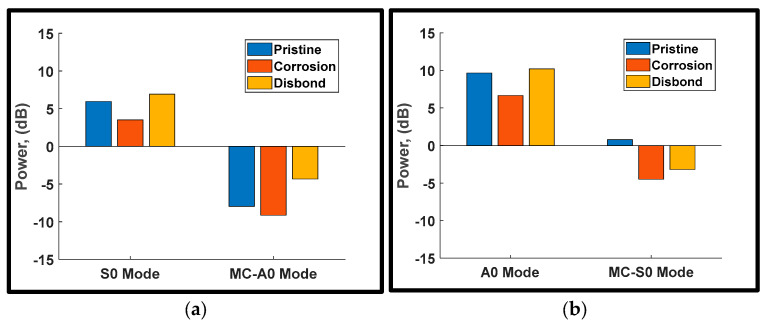
Power losses of Lamb mode incidents and converted modes using a five-cycle Hann windowed excitation signal at 400 kHz center frequency: (**a**) S0 incident; and (**b**) A0 incident.

Figure 17b shows the power losses of the transmitted A0 incident and MC-S0 for the pristine and damaged conditions. Another distinctive signature was noticed from monitoring MC-S0. This converted mode experienced a gain of about 3 dB due to the presence of damage, in contrast to its power loss of 0.899 dB in the pristine state. The same trend was also observed at a higher actuation frequency of 400 kHz as shown in Figure 18. The results suggest that both corrosion and disbonding yield a positive gain by more than 3 dB for the MC-S0 mode, providing an effective signature for damage detection without relying on baseline signals.

Furthermore, corrosion and disbonding can be distinguished by monitoring the transmission losses of Lamb mode incidents and converted modes. By careful inspection of the results in Figure 17 and Figure 18, the presence of corrosion can be identified by the power gain in converted modes and the drop in the transmission losses of Lamb mode incidents. On the other hand, disbonding can be clearly detected by the substantial loss of the S0 incident and the positive gain in converted modes, particularly the MC-S0 mode.

## 4. Conclusions

This study investigates the propagation of guided waves in steel welded lap joints for baseline-free inspections of joint defects using scattering and the mode conversion of Lamb waves. The guided waves were simulated using the finite element method. The simulation model was validated by comparing the dispersion curves, mode shapes, and transmission losses of guided waves with analytical measurements and existing literature data. Two types of joint defects including corrosion and disbonding were inflicted in the welded lap joint to detect damage. To analyze the characteristics of guided waves, a wavenumber analysis was conducted on both the upstream and downstream signals across the lap joint using the 2D FFT technique. The 2D FFT was employed for 80 signals collected at equidistant points defined before and after the lap joints. The following conclusions were drawn from the study:

- Welded lap joints have resulted in scattered and superposed wave modes when fundamental Lamb modes were excited in pristine conditions. The A0 incident experienced significant transmission losses of 9.46 dB compared to a slight attenuation of 2.89 dB for the S0 incident. The antisymmetric actuation, however, provided higher resolution downstream signals in comparison to the symmetric actuation due to the dominance of the A0 mode in the medium.
- The presence of corrosion in lap joints reduced transmission losses by more than 28% for both the S0 and A0 incidents. The results suggest that using the A0 incident to determine the corrosion in a pitch-catch arrangement can potentially improve the signal resolution and consequently reduce the complexity of signal processing.
- Introducing disbonding in the lap joint has significant effects on the S0 incident, leading to a transmission loss of 7.36 dB, while relatively negligible losses are observed for the A0 incident. Unique modes, such as RF-S0 and MC-A0, were also noticed in the upstream signals, making the use of the S0 incident more attractive to determine disbonding.
- Monitoring MC-A0 can essentially be used as a signature for detecting the presence of corrosion or disbonding without relying on baseline signals. This converted mode exhibited a noticeable gain in the range of 5 dB to 11 dB in the simulated damage scenarios.
- Another distinctive signature was observed from monitoring the MC-S0 which experienced a gain due to the presence of corrosion and disbonding, in contrast to the power loss observed in the pristine state. These signatures can potentially be used to assist in identifying the type of flaw in the welded lap joints.

The results indicate that Lamb mode incidents and converted modes interact differently with corrosion and disbonding, providing a means to determine damage without relying on baseline signals. Such trends could potentially be leveraged to distinguish between corrosion and disbonding. This work is expected to be of importance in the field of the nondestructive evaluation of welded lap joints. Thus, experimental work with different types of joint defects, including corrosion and disbonding, should be performed to validate the analysis outcomes of numerical simulations presented herein. The dispersion effects of guided Lamb waves propagating through welded lap joints should be taken into consideration to reduce the uncertainty of baseline-free inspections. Operational and environmental conditions could have pronounced effects on guided waves and consequently on signal processing. Future studies should incorporate such effects in numerical simulations and experiments to improve non-destructive evaluation outcomes.

## Figures and Tables

**Table 1:** Group velocities and power losses of the S0 incident and MC-A0 in pristine condition.

Mode	$\mathrm{Analytical}\;\mathrm{Group}\;\mathrm{Velocity},\;v_{gr}$ (mm/μs)	$\mathrm{Numerical}\;\mathrm{Group}\;\mathrm{Velocity},\;v_{gr}$ (mm/μs)	Numerical Power Loss, α(dB)	Numerical Power Loss, α(dB) [23]
S0	5.275	5.128	2.89	4.3
MC-A0	2.580	2.513	------	-----

**Table 2:** Group velocities and power losses of the A0 incident and MC-S0 in pristine condition.

Mode	$\mathrm{Analytical}\;\mathrm{Group}\;\mathrm{Velocity},\;v_{gr}$(mm/μs)	$\mathrm{Numerical}\;\mathrm{Group}\;\mathrm{Velocity},\;v_{gr}$(mm/μs)	Numerical Power Loss, α (dB)
A0	2.580	2.645	9.46
MC-S0	5.275	5.467	----

## Data Availability

Data are contained within the article.
